# The ethnicity-specific association of biomarkers with the angiographic severity of coronary artery disease

**DOI:** 10.1007/s12471-015-0798-y

**Published:** 2016-01-11

**Authors:** C. M. Gijsberts, A. Seneviratna, I. E. M. Bank, H. M. den Ruijter, F. W. Asselbergs, P. Agostoni, J. A. Remijn, G. Pasterkamp, H. C. Kiat, M. Roest, A. M. Richards, M. Y. Chan, D. P. V. de Kleijn, I. E. Hoefer

**Affiliations:** 1 Laboratory of Experimental Cardiology, University Medical Center Utrecht, Utrecht, The Netherlands; 2 The Netherlands Heart Institute (ICIN), Utrecht, The Netherlands; 3 Cardiac Department, National University Heart Centre, National University Hospital, Singapore, Singapore; 4 Cardiology, University Medical Center Utrecht, Utrecht, The Netherlands; 5 Durrer Center for Cardiogenetic Research, ICIN-Netherlands Heart Institute, Utrecht, The Netherlands; 6 Institute of Cardiovascular Science, faculty of Population Health Sciences, University College London, London, United Kingdom; 7 Clinical Chemistry and Hematology, Gelre Hospitals, Apeldoorn, The Netherlands; 8 Clinical Chemistry and Hematology, University Medical Center Utrecht, Utrecht, The Netherlands; 9 Department of Paediatrics, National University of Singapore, Singapore, Singapore; 10 Cardiovascular Research Institute, National University Heart Centre, National University Health System, Singapore, Singapore

**Keywords:** Ethnicity, Coronary angiography, Biomarkers, Singapore

## Abstract

**Background:**

Risk factor burden and clinical characteristics of patients with coronary artery disease (CAD) differ among ethnic groups. We related biomarkers to CAD severity in Caucasians, Chinese, Indians and Malays.

**Methods:**

In the Dutch-Singaporean UNICORN coronary angiography cohort (*n* = 2033) we compared levels of five cardiovascular biomarkers: N-terminal pro-brain natriuretic peptide (NTproBNP), high-sensitivity C-reactive protein (hsCRP), cystatin C (CysC), myeloperoxidase (MPO) and high-sensitivity troponin I (hsTnI). We assessed ethnicity-specific associations of biomarkers with CAD severity, quantified by the SYNTAX score.

**Results:**

Adjusted for baseline differences, NTproBNP levels were significantly higher in Malays than in Chinese and Caucasians (72.1 vs. 34.4 and 41.1 pmol/l, *p* < 0.001 and *p* = 0.005, respectively). MPO levels were higher in Caucasians than in Indians (32.8 vs. 27.2 ng/ml, *p* = 0.026), hsTnI levels were higher in Malays than in Caucasians and Indians (33.3 vs. 16.4 and 17.8 ng/l, *p* < 0.001 and *p* = 0.029) and hsTnI levels were higher in Chinese than in Caucasians (23.3 vs. 16.4, *p* = 0.031). We found modifying effects of ethnicity on the association of biomarkers with SYNTAX score. NTproBNP associated more strongly with the SYNTAX score in Malays than Caucasians (β 0.132 vs. β 0.020 per 100 pmol/l increase in NTproBNP, *p* = 0.032). For MPO levels the association was stronger in Malays than Caucasians (β 1.146 vs. β 0.016 per 10 ng/ml increase, *p* = 0.017). Differing biomarker cut-off levels were found for the ethnic groups.

**Conclusion:**

When corrected for possible confounders we observe ethnicity-specific differences in biomarker levels. Moreover, biomarkers associated differently with CAD severity, suggesting that ethnicity-specific cut-off values should be considered.

## Introduction

Coronary artery disease (CAD) is highly prevalent worldwide but over the next few decades the majority of deaths due to CAD will occur in Asia [1]. Evidence is accumulating that important differences exist between the Asian ethnic groups and Caucasians, who have been the main focus of cardiovascular research up until now. We know that risk factor levels differ markedly. Specifically, a high prevalence of diabetes has been described for South Asians [2]. Also, the CAD phenotype appears to differ among the ethnic groups. Triple-vessel disease is more common in South Asians than in Caucasians [3], whilst the Chinese suffer from less severe CAD [4].

Blood-derived biomarkers are noninvasive tools that can be indicative of CAD severity. Population means of biomarker levels differ among ethnic groups [5]. However, it is unknown whether biomarkers of CAD report similarly on underlying CAD severity among these groups [6]. In the current study, we investigated patients undergoing coronary angiography for suspected CAD from four globally populous ethnic groups: Caucasians, Chinese, Indians and Malays, enrolled in two countries with high, comparable healthcare standards [7]: the Netherlands and Singapore. We evaluated five established biomarkers known to be affected by CAD: N-terminal pro-brain natriuretic peptide (NTproBNP) [8–10], high-sensitivity C-reactive protein (hsCRP) [11], cystatin C (CysC) [12, 13], myeloperoxidase (MPO) [14, 15] and high-sensitivity troponin I (hsTnI) [16, 17]. These biomarkers reflect pivotal CAD aspects and complications: cardiac haemodynamic load (NTproBNP), inflammation (hsCRP and MPO), kidney function (CysC) and cardiomyocyte damage (hsTnI).

In this study we aimed to define inter-ethnic differences in the association of these biomarkers with CAD severity, quantified by the SYNTAX score [18].

## Methods

This study was conducted using the parallel United CORoNary biobanks, the UNICORN cohort (clinicaltrials.gov ID: NCT02126150), consisting of consecutive patients undergoing coronary angiography recruited from two sites: the University Medical Center Utrecht, the Netherlands and the National University Hospital, Singapore. The institutional review boards of both centres approved of this study, which conforms to the declaration of Helsinki.

Patients were enrolled between September 2010 and March 2013. Patients from four ethnic groups were enrolled, namely Caucasians in the Netherlands and Chinese, Indians and Malays in Singapore. Blood was sampled from the arterial sheath, inserted at commencement of the coronary angiography procedure and immediately stored at − 80 °C.

### Clinical and angiographic characteristics

Risk factor profiles were documented at or around the time of coronary angiography. Coronary angiograms were categorised into four groups: no/minor CAD, single-vessel disease, double-vessel disease and triple-vessel disease (defined as number of epicardial vessels with 50 % stenosis [19] on visual assessment). In the last three categories, a SYNTAX score was determined [18]. The process of SYNTAX scoring has been described previously [20].

### Biomarker assays

Plasma levels of NTproBNP, hsCRP, CysC and MPO were measured at the University Medical Center Utrecht, the Netherlands, using a semi-automated ELISA robot (Freedom EVO, Tecan, Switzerland). Commercial antibody combinations were used to quantify NTproBNP (15 C4 and biotinylated 13G12, Hi-test Finland), hsCRP (Dy1707 duoset, R&D systems), CysC (Dy1196 duoset, R&D systems) and MPO (Dy3667 duoset, R&D systems). In brief, maxisorb plates were coated with mouse anti-human NTproBNP, hsCRP, CysC or MPO. Plates were blocked with 1 % bovine serum albumin, and incubated with supernatants. Plates were washed with phosphate buffered saline pH 7.4 with 0.05 % Tween 20. Bound factors were detected with biotin coupled detection antibodies. Biotin coupled antibodies were bound with streptavidin horseradish peroxidase (HRP), or goat-anti-human antibodies with rabbit-anti-goat HRP (DAKO, P0449). Detection was performed with SuperSignal West Pico Chemiluminescent substrate, and read with a luminometer. The intra-assay variation coefficient was 10 %. Levels of hsTnI were measured using the *STAT* hsTn1I assay on the clinically validated ARCHITECT *i*2000 analyser (Abbott Laboratories, Lisnamuck, Longford, Ireland).

All samples (Singaporean and Dutch) were randomly distributed across the plates for all analyses.

### Statistical analysis

Figures are presented as means with standard deviations for normally distributed variables or medians with interquartile ranges for non-normally distributed variables. Baseline characteristics were compared among the ethnic groups using ANOVA for normally distributed variables, Kruskal-Wallis tests for non-normally distributed data and chi-square tests for categorical data.

We evaluated inter-ethnic differences in biomarker levels, adjusted for baseline differences in age, sex, body mass index, diabetes, hypertension, dyslipidaemia, smoking, indication for procedure, severity of CAD, antiplatelet medication use, beta blocker use, calcium antagonist use, renin-angiotensin-aldosterone system medication use and statin use. Using ANCOVA, adjusted biomarker levels were calculated for each ethnic group. Biomarker levels were positively skewed and therefore log-transformed for analysis when used as the dependent variable. The presented adjusted biomarker level means are antilogs (back-transformed after analysis). Biomarker levels were compared among the ethnic groups through Tukey post-hoc testing, thus adjusting for multiple testing.

Also, we tested for interactions between ethnicity and biomarker levels for the SYNTAX score in univariable and multivariable regression models. Direct comparison of biomarker coefficients between ethnicities was performed by testing an interaction term of biomarker level with ethnicity; Caucasian ethnicity was the reference group. A conservative p-value of 0.05 for the interaction terms was deemed significant.

Next, using receiver operating characteristic (ROC) analysis, we compared the optimal cut-off biomarker values by ethnicity, defined as the biomarker level at which sensitivity + specificity for a high SYNTAX score was largest. Also, areas under curves were calculated for association with a high SYNTAX score for each biomarker in each ethnic group [21]. The outcome SYNTAX score was dichotomised on a previously reported [22] cut-off of 18 points.

All analyses were performed using the R software package [23]. A two-tailed α of: 0.05 was considered statistically significant.

## Results

### Baseline characteristics

Marked baseline differences were observed among the ethnic groups (Table 1). Indian and Malay patients were younger (54.4 and 55.4 years, respectively) than the Chinese and Caucasian patients (59.1 and 64.7 years, respectively, *p* < 0.001). Among Indian and Malay patients, diabetes (54.4 and 52.5 %, respectively) and dyslipidaemia (76.6 and 75.1 %, respectively) were strikingly more common than in Caucasians (diabetes 22.6 %, *p* < 0.001, dyslipidaemia 48.2 %, *p* < 0.001). The prevalence of current smokers was highest among Indians (42.9 %).

**Table 1:** Baseline characteristics of UNICORN participants, stratified by ethnicity

	Caucasian	Chinese	Indian	Malay	*p*-value
*N*	1132	562	158	181	
Males (%)	72.5	81.0	80.4	79.6	< **0.001**
Age (mean (SD), years)	64.7 (11.3)	59.1 (9.9)	54.4 (9.5)	55.4 (9.4)	< **0.001**
BMI (mean (SD), kg/m^2^)	27.0 (4.3)	26.2 (4.7)	27.5 (5.0)	28.5 (5.4)	< **0.001**
Diabetes (%)	22.6	34.0	54.4	52.5	< **0.001**
Hypertension (%)	56.1	65.1	63.9	64.6	**0.001**
Dyslipidaemia (%)	48.2	69.6	76.6	75.1	< **0.001**
Smoking, current (%)	23.6	29.4	42.9	39.0	< **0.001**
Smoking, ex (%)	31.6	19.2	11.9	16.4	
Renal failure (%)	3.1	6.0	3.8	5.6	**0.034**
*Medications*					
Platelet inhibitor (%)	64.6	58.4	57.6	55.8	**0.016**
Statin (%)	58.9	44.3	43.0	43.1	< **0.001**
Beta blocker (%)	25.4	26.7	24.7	19.3	0.259
RAAS (%)	51.4	35.2	40.5	43.6	< **0.001**
Calcium antagonist (%)	64.3	61.4	61.4	55.8	0.146
*Medical history*					
Previous PCI (%)	31.6	17.5	29.1	30.0	< **0.001**
Previous CABG (%)	13.0	6.2	6.3	6.1	< **0.001**
Previous ACS (%)	32.6	14.8	26.8	28.5	< **0.001**
History of CVA/TIA (%)	10.3	5.7	8.2	6.7	**0.010**
History of PAD (%)	11.5	2.8	4.4	5.0	< **0.001**
*Indication for coronary angiography*					
Stable CAD (%)	67.4	65.9	58.8	54.8	< **0.001**
UA/NSTEMI (%)	22.5	31.6	36.6	42.9	
STEMI (%)	10.1	2.4	4.6	2.3	
*CAD severity*					
No/minor CAD (%)	25.0	33.8	28.7	25.1	< **0.001**
Single-vessel disease (%)	37.3	21.7	24.0	21.1	
Double-vessel disease (%)	25.5	23.6	25.3	27.4	
Triple-vessel disease (%)	12.2	20.8	22.0	26.3	
*Treatment*					
Conservative (%)	35.0	53.6	58.2	55.2	< **0.001**
PCI (%)	58.8	37.5	37.3	33.7	
CABG (%)	6.2	8.9	4.4	11.0	
*Biomarker levels*					
NTproBNP (median [IQR])	43.1 [10.5, 138.0]	37.8 [11.1, 135.2]	31.5 [8.2, 108.0]	63.9 [16.3, 201.8]	**0.049**
hsCRP (median [IQR])	1.5 [0.6, 3.8]	1.3 [0.6, 4.2]	2.3 [1.0, 6.7]	2.2 [0.8, 7.3]	< **0.001**
CysC (median [IQR])	0.8 [0.7, 1.1]	0.7 [0.6, 0.9]	0.8 [0.6, 0.9]	0.8 [0.5, 1.0]	< **0.001**
MPO (median [IQR])	28.1 [20.7, 44.8]	27.0 [20.6, 37.0]	26.8 [19.8, 35.1]	30.3 [23.5, 40.9]	**0.001**
hsTnI (median [IQR])	7.8 [3.8, 28.4]	8.1 [4.2, 48.1]	7.7 [4.2, 69.1]	12.2 [4.6, 209.5]	**0.001**
*Outcome*					
SYNTAX score (mean (SD))	11.6 (8.2)	15.2 (10.0)	14.5 (10.6)	15.6 (11.3)	< **0.001**
High SYNTAX score (n (%) ≥ 18 points)	147 (21.0)	116 (34.0)	34 (33.0)	47 (38.2)	< **0.001**
FU time (median [IQR], days)	823 [653, 988]	930 [734, 1062]	882 [714, 1044]	852 [649, 1045]	< **0.001**
All-cause death (n)	75	36	3	11	

Baseline characteristics of UNICORN patients stratified by ethnicity. Non-normally distributed continuous variables were compared using ANOVA across ethnic groups. Biomarker levels were compared by Kruskal-Wallis testing. Categorical data were compared using chi-square testing. Reported *p*-values refer to overall differences across the ethnic groups. Significant *p*-values are printed in bold.

*BMI* body mass index, *RAAS* renin-angiotensin-aldosterone system, *PCI* percutaneous coronary intervention, *CABG* coronary artery bypass grafting, *ACS* acute coronary syndrome, *CVA* cerebrovascular accident, *TIA* transient ischaemic attack, *PAD* peripheral artery disease, *CAD* coronary artery disease, *UA* unstable angina, *NSTEMI* non-ST-elevated myocardial infarction, *STEMI* ST-elevated myocardial infarction, *NTproBNP* N-terminal brain natriuretic peptide, *hsCRP* high-sensitivity C-reactive protein, *CysC* Cystatin C, *MPO* myeloperoxidase, *hsTnI* high-sensitivity troponin I, *FU* time follow-up time.

The indication for angiography differed among the ethnic groups (*p* < 0.001). Stable CAD was slightly less common among Indians and Malays (58.8 and 54.8 %, respectively, vs. 67.4 % in Caucasians and 65.9 % in Chinese). Unstable angina or non-ST-elevated myocardial infarction was more frequently observed in all three Asian groups than in Caucasians (Chinese 31.6 %, Indians 36.6 %, Malays 42.9 vs. 22.5 % in Caucasians, *p* < 0.001). Also, triple-vessel disease was more often diagnosed in the Asian ethnic groups than in Caucasians (Chinese 20.8 %, Indian 22.0 %, Malay 26.3 vs. 12.2 % in Caucasians, *p* < 0.001).

Percutaneous coronary intervention (PCI) was performed more often on Caucasians than in the other ethnic groups, in whom a conservative strategy was more frequently opted for (*p* < 0.001). Coronary artery bypass graft (CABG) surgery was performed most often in Malays (11.0 vs. 4.4 % in Indians, 6.2 % in Caucasians and 8.9 % in Chinese).

### Biomarker levels

**Fig. 1:**
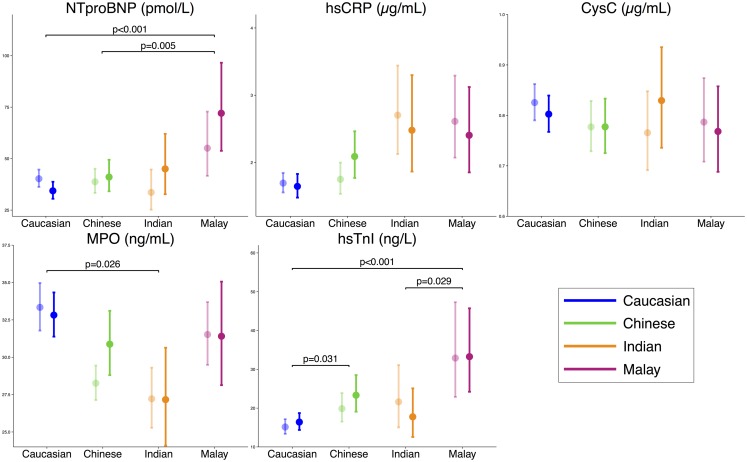
Biomarker levels by ethnicity, corrected for baseline differences (ANCOVA). The unadjusted means of biomarker levels (transparent) and adjusted means derived from ANCOVA (solid) are shown. The adjusted biomarker levels are corrected for: age, sex, BMI, diabetes, hypertension, dyslipidaemia, smoking, indication for angiography, CAD severity, antiplatelet medication use, beta blocker use, calcium antagonist use, RAAS inhibiting medication use and statin use. CysC levels were additionally corrected for renal failure. *P*-values that are presented in the plots are the result of ANCOVA with post-hoc testing (*p*-values are corrected according to the Tukey method)

Crude and multivariable-adjusted biomarker levels are displayed in Fig. 1, stratified by ethnicity. Crude biomarker levels differed significantly among the ethnic groups for all of the examined biomarkers (Table 1). In Table 2, the crude biomarker levels are displayed for each ethnic group, stratified by indication for angiography and angiographic severity of CAD. All biomarkers differed among the ethnic groups in at least one indication group. High-sensitivity TnI did not differ in any of the CAD severity groups, while the other biomarkers did differ significantly in at least one CAD severity group.

**Table 2:** Biomarker levels stratified by indication for angiography and by CAD severity, for each ethnicity

Biomarker		Caucasian	Chinese	Indian	Malay	*p*-value
*Indication*						
**NTproBNP (pmol/l)**	Stable CAD	42.7 [13.41, 133.96]	25.71 [4.04, 60.78]	22.50 [6.95, 52.11]	40.35 [15.81, 157.66]	< **0.001**
	UA/NSTEMI	48.86 [13.29, 162.27]	77.99 [25.47, 257.85]	49.85 [6.78, 255.63]	70.92 [18.95, 289.93]	**0.041**
	STEMI	21.64 [3.00, 77.01]	122.87 [55.78, 446.12]	119.65 [103.66, 635.87]	274.25 [206.61, 2245.84]	< **0.001**
**hsCRP (μg/ml)**	Stable CAD	1.35 [0.55, 3.10]	1.01 [0.46, 2.58]	1.92 [0.98, 3.74]	1.70 [0.70, 5.00]	< **0.001**
	UA/NSTEMI	2.11 [0.74, 5.88]	3.40 [0.80, 11.36]	3.10 [1.31, 8.81]	3.65 [1.27, 10.33]	0.070
	STEMI	1.95 [0.60, 5.20]	9.42 [1.39, 19.96]	28.46 [8.97, 61.99]	21.25 [12.46, 31.37]	**0.002**
**CysC (μg/ml)**	Stable CAD	0.84 [0.66, 1.08]	0.70 [0.58, 0.87]	0.73 [0.57, 0.88]	0.76 [0.53, 0.98]	< **0.001**
	UA/NSTEMI	0.83 [0.65, 1.07]	0.79 [0.65, 1.02]	0.80 [0.64, 1.05]	0.81 [0.59, 1.07]	0.894
	STEMI	0.75 [0.59, 0.90]	0.76 [0.64, 0.87]	0.68 [0.62, 0.86]	0.55 [0.48, 0.75]	0.809
**MPO (ng/ml)**	Stable CAD	24.93 [19.29, 33.05]	25.21 [19.41, 33.61]	26.8 [19.73, 32.45]	28.83 [22.51, 38.81]	0.071
	UA/NSTEMI	32.86 [22.94, 55.28]	30.74 [23.50, 41.80]	24.98 [20.14, 36.6]	35.7 [24.98, 45.22]	**0.011**
	STEMI	137.68 [62.42, 210.34]	35.39 [28.93, 37.93]	27.89 [24.03, 34.51]	28.7 [22.35, 37.85]	< **0.001**
**hsTnI (ng/l)**	Stable CAD	5.60 [3.30, 12.03]	5.60 [3.50, 11.50]	4.70 [3.50, 13.5]	8.30 [4.00, 18.8]	**0.034**
	UA/NSTEMI	26.10 [7.00, 283.1]	102.65 [14.80, 1153.83]	36.35 [7.15, 229.38]	171.30 [8.25, 1249.40]	**0.002**
	STEMI	128.55 [26.43, 944.68]	78.70 [20.10, 2750.00]	4065.10 [1651.45, 21301.05]	1206.25 [956.28, 2135.23]	**0.005**
*CAD severity*						
**NTproBNP (pmol/l)**	No/minor CAD	39.83 [8.64, 132.79]	24.48 [3.00, 80.88]	8.39 [3.00, 32.06]	22.71 [3.00, 97.30]	**0.002**
	Single-vessel disease	40.3 [11.75, 123.41]	27.39 [7.72, 68.81]	34.16 [15.73, 84.92]	34.38 [5.00, 107.95]	0.351
	Double-vessel disease	39.48 [7.31, 159.11]	44.86 [19.34, 155.28]	38.43 [16.08, 105.24]	66.45 [18.36, 311.30]	0.315
	Triple-vessel disease	68.93 [22.53, 164.18]	70.98 [24.12, 233.65]	61.61 [15.51, 397.78]	162.41 [35.96, 414.94]	0.079
**hsCRP (μg/ml)**	No/minor CAD	1.20 [0.55, 3.11]	1.10 [0.51, 3.30]	2.66 [1.31, 7.67]	1.93 [0.69, 6.17]	**0.002**
	Single-vessel disease	1.41 [0.62, 3.47]	1.05 [0.48, 3.01]	2.58 [1.02, 4.51]	2.19 [1.29, 8.79]	**0.001**
	Double-vessel disease	1.47 [0.57, 3.84]	1.51 [0.60, 5.58]	1.78 [0.85, 3.67]	2.74 [0.82, 8.29]	0.235
	Triple-vessel disease	2.51 [0.80, 5.26]	1.89 [0.76, 8.28]	2.70 [1.00, 9.81]	2.15 [0.90, 6.93]	0.822
**CysC (μg/ml)**	No/minor CAD	0.80 [0.65, 1.02]	0.70 [0.58, 0.88]	0.72 [0.62, 0.87]	0.63 [0.51, 0.93]	< **0.001**
	Single-vessel disease	0.82 [0.64, 1.05]	0.71 [0.56, 0.84]	0.68 [0.60, 0.85]	0.67 [0.54, 0.81]	< **0.001**
	Double-vessel disease	0.86 [0.68, 1.09]	0.74 [0.60, 0.95]	0.75 [0.62, 0.89]	0.87 [0.57, 1.08]	**0.010**
	Triple-vessel disease	0.85 [0.69, 1.07]	0.77 [0.63, 0.94]	0.86 [0.63, 1.19]	0.88 [0.60, 1.14]	0.266
**MPO (ng/ml)**	No/minor CAD	26.00 [19.99, 37.21]	25.34 [18.68, 33.80]	28.90 [21.14, 32.72]	29.33 [22.55, 38.88]	0.272
	Single-vessel disease	28.01 [21.06, 42.76]	29.36 [21.40, 40.95]	27.53 [18.12, 37.14]	29.53 [24.11, 41.49]	0.516
	Double-vessel disease	27.87 [20.63, 46.29]	27.07 [21.13, 37.54]	23.57 [20.05, 32.82]	28.19 [23.15, 40.79]	0.183
	Triple-vessel disease	30.92 [22.88, 74.03]	29.57 [21.91, 40.13]	25.80 [19.71, 35.89]	35.24 [25.66, 40.83]	**0.033**
**hsTnI (ng/l)**	No/minor CAD	5.25 [2.80, 12.5]	5.10 [3.30, 12.08]	5.00 [3.10, 15.60]	9.15 [3.60, 19.53]	0.200
	Single-vessel disease	7.10 [3.70, 30.6]	7.20 [4.00, 54.8]	14.75 [3.90, 123.50]	5.90 [3.50, 20.20]	0.437
	Double-vessel disease	10.50 [4.80, 39.85]	12.25 [4.53, 109.1]	7.25 [4.60, 62.73]	13.80 [5.75, 825.80]	0.187
	Triple-vessel disease	18.30 [6.60, 228.30]	33.55 [7.88, 398.03]	29.10 [7.00, 299.30]	70.30 [10.50, 776.80]	0.115

Medians and interquartile ranges of biomarker levels, stratified by indication for angiography and by angiographic CAD severity. *P*-values for comparison among the ethnic groups are from Kruskal-Wallis testing. Significant *p*-values are printed in bold.

*CAD* coronary artery disease, *UA* unstable angina, *NSTEMI* non-ST-elevation myocardial infarction, *STEMI* ST-elevation myocardial infarction, *NTproBNP* N-terminal pro brain natriuretic peptide, *hsCRP* high-sensitivity C-reactive protein, *CysC* cystatin C, *MPO* myeloperoxidase, *hsTnI* high-sensitivity troponin I

When adjusted for baseline characteristics, certain differences in biomarker levels remained statistically significant. Post-hoc testing revealed that multivariable-adjusted NTproBNP levels were significantly higher in Malays than in Chinese and Caucasians (72.1 vs. 34.4 and 41.1 pmol/l, *p* < 0.001 and *p* = 0.005, respectively). MPO levels were higher in Caucasians than in Indians (32.8 vs. 27.2 ng/ml, *p* = 0.026), hsTnI levels were higher in Malays than in Caucasians and Indians (33.3 vs. 16.4 and 17.8 ng/l, *p* < 0.001 and *p* = 0.029) and hsTnI levels were higher in Chinese than in Caucasians (23.3 vs. 16.4, *p* = 0.031). High-sensitivity CRP levels and CysC levels (additionally adjusted for renal failure) did not differ among the ethnic groups.

### Modifying effect of ethnicity on association between biomarker levels and SYNTAX score

**Fig. 2:**
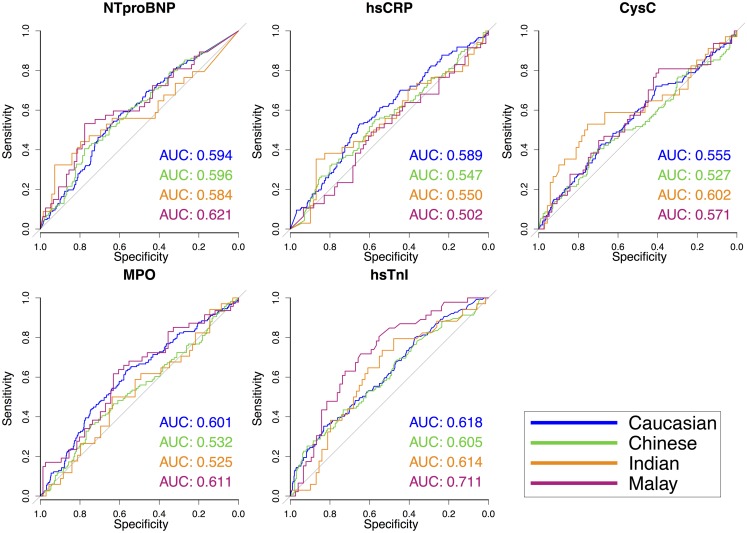
Ethnicity-specific association of biomarker levels with SYNTAX score from univariable regression model. Significant interactions (*p* < 0.05) were found in a multivariable model for: NTproBNP with Malay ethnicity (*p* = 0.032) and MPO with Malay ethnicity (*p* = 0.017) as compared with Caucasian ethnicity

We tested interactions of ethnicity with biomarker levels for SYNTAX score (univariable and multivariable, Table 3 and Fig. 2). We found a significantly higher beta for NTproBNP levels in Malays than in Caucasians (only in the multivariable model, β 0.132 vs. β 0.020 *p* = 0.032), indicating a steeper increase in SYNTAX score with every 100-unit increase of NTproBNP in Malays than in Caucasians. Also, we found a significantly higher beta, in both the univariable (β 1.517 vs. β 0.101, *p* = 0.002) and the multivariable (β 1.146 vs. β 0.016, *p* = 0.017) model, for MPO levels in Malays than in Caucasians, indicating that with a 10-unit increase of MPO, the SYNTAX score increases more steeply in Malays than in Caucasians. For hsTnI levels (per 100-unit increase) we found a lower beta for Malays than for Caucasians (β 0.003 vs. β 0.044, *p* = 0.010); however, this difference was abolished when adjusting for baseline differences.

**Table 3:** Regression coefficients (betas with 95 % confidence intervals) of biomarker levels for SYNTAX score

Biomarker	Ethnicity	Beta (CI, univariable)	*p*-value beta (univ.)	Beta (CI, multivariable)	*p*-value beta (multiv.)	*p*-value interaction (univ.)	*p*-value interaction (multiv.)
**NTproBNP (pmol/l)** ^a^	Caucasian	0.056 (− 0.002–0.114)	0.059	0.020 (− 0.047–0.087)	0.560	Ref.	Ref.
	Chinese	0.153 (0.053–0.254)	**0.003**	0.104 (− 0.013–0.221)	0.081	0.094	0.115
	Indian	0.088 (− 0.065–0.241)	0.255	0.020 (− 0.119–0.160)	0.771	0.669	0.361
	Malay	0.152 (0.011–0.292)	**0.035**	0.132 (− 0.020–0.284)	0.088	0.157	**0.032**
**hsCRP (μg/ml)**	Caucasian	0.041 (0.009–0.074)	**0.012**	0.030 (− 0.006–0.066)	0.103	Ref.	Ref.
	Chinese	0.029 (− 0.020–0.077)	0.243	0.035 (− 0.025–0.095)	0.255	0.663	0.709
	Indian	0.009 (− 0.095–0.112)	0.870	0.033 (− 0.084–0.150)	0.578	0.505	0.627
	Malay	− 0.025 (− 0.117–0.066)	0.584	− 0.040 (− 0.148–0.069)	0.471	0.113	0.621
**CysC (μg/ml)**	Caucasian	0.162 (− 0.015–0.340)	0.073	0.081 (− 0.131–0.293)	0.454	Ref.	Ref.
	Chinese	0.235 (− 0.015–0.485)	0.066	0.154 (− 0.194–0.503)	0.383	0.642	0.371
	Indian	0.022 (− 0.449–0.492)	0.928	− 0.172 (− 0.700–0.356)	0.519	0.541	0.753
	Malay	− 0.168 (− 0.923–0.586)	0.660	− 0.165 (− 0.935–0.605)	0.671	0.314	0.457
**MPO (ng/ml)** ^b^	Caucasian	0.101 (0.015–0.187)	**0.022**	0.016 (− 0.095–0.127)	0.777	Ref.	Ref.
	Chinese	− 0.008 (− 0.485–0.469)	0.974	− 0.079 (− 0.630–0.472)	0.778	0.633	0.885
	Indian	− 0.077 (− 1.314–1.160)	0.902	0.108 (− 1.099–1.314)	0.859	0.743	0.555
	Malay	1.517 (0.451–2.583)	**0.006**	1.146 (0.023–2.269)	**0.046**	**0.002**	**0.017**
**hsTnI (ng/l)** ^a^	Caucasian	0.044 (0.026–0.062)	< **0.001**	0.032 (0.012–0.052)	**0.002**	Ref.	Ref.
	Chinese	0.035 (0.008–0.061)	**0.010**	0.044 (0.013–0.074)	**0.006**	0.557	0.164
	Indian	0.022 (− 0.023–0.067)	0.331	0.050 (− 0.004–0.104)	0.067	0.328	0.958
	Malay	0.003 (− 0.027–0.033)	0.858	0.003 (− 0.027–0.033)	0.855	**0.010**	0.351

The multivariable model adjusts for: age, sex, BMI, diabetes, hypertension, hyperlipidaemia, smoking status, indication for coronary angiography, antiplatelet medication use, beta blocker use, calcium antagonist use, RAAS medication use and statin use. Interaction terms were tested in the univariable and the multivariable model. Ref. = reference group for interaction analysis.

*CI* 95 % confidence interval, *univ*. univariable, multiv. multivariable, *NTproBNP* N-terminal pro brain natriuretic peptide, *hsCRP* high-sensitivity C-reactive protein, *CysC* Cystatin C, *MPO*  myeloperoxidase, *hsTnI* high-sensitivity troponin I. Significant p-values are printed in bold.

^a^Betas are displayed for a 100-unit increase in biomarker level.

^b^Betas are given for a 10-unit increase in MPO levels.

### Biomarker discrimination of high SYNTAX score

**Fig. 3:**
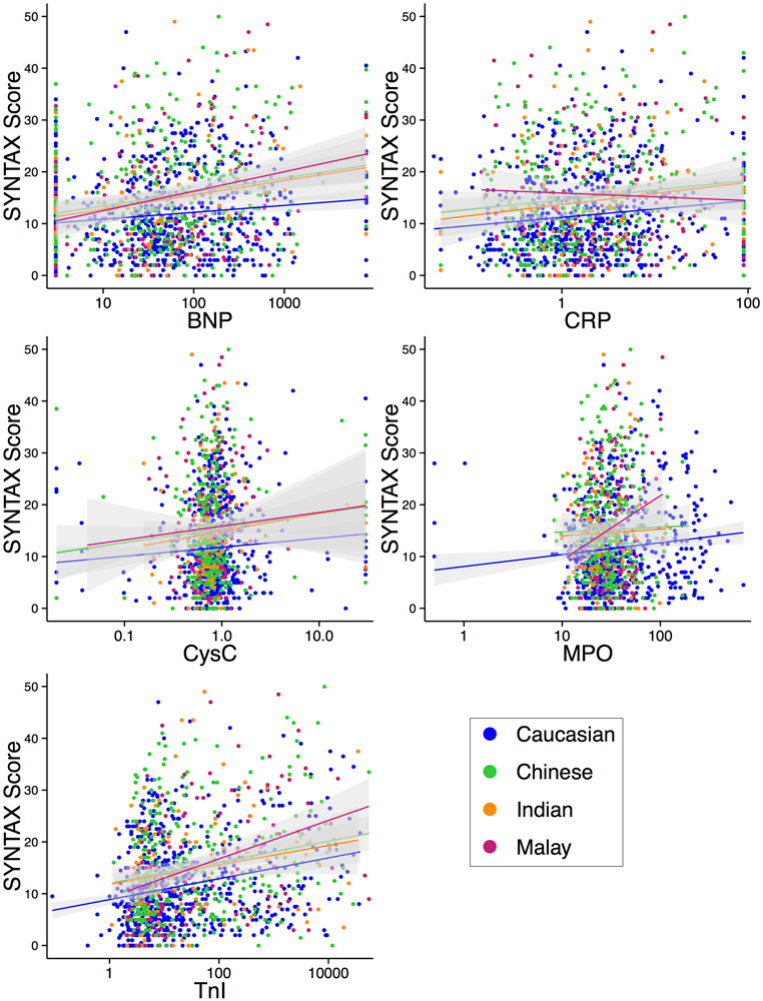
Receiver operating characteristic (ROC) curves with area under the curves (AUC) of biomarker levels for high SYNTAX score (≥ 18 points). ROC curves and AUCs of biomarker level performance for each ethnic group. No significant differences between the AUCs were found for any of the biomarkers. The diagonal line represents an AUC of 0.5. The further a line deviates to the upper left, the better the discriminating properties (higher sensitivity and specificity) for a SYNTAX score of ≥ 18 points

From ROC analysis (Fig. 3) we determined ethnicity-specific optimal biomarker cut-off values for the association with a SYNTAX score of ≥ 18. These cut-offs (Table 4) correspond with the biomarker level at which the sum of sensitivity + specificity is largest. We found markedly different biomarker cut-off values across the ethnic groups. The optimal NTproBNP cut-off for Indians, for example, was 5-fold higher than for Caucasians: 255.77 pmol/l vs. 48.42 pmol/l. For hsCRP the cut-off was highest among Indians (8.95 μg/ml) and lowest in Chinese (1.69 μg/ml). The cut-offs for CysC and MPO were in the same order of magnitude among the ethnic groups.

**Table 4:** Results from ROC analysis of biomarker levels for high SYNTAX score (≥ 18 points)

Biomarker	Ethnicity	Optimal cut-off	AUC (95 % CI)
**NTproBNP (pmol/l)**	Caucasian	48.42	**0.594 (0.543–0.645)**
	Chinese	120.92	**0.596 (0.532–0.659)**
	Indian	255.77	0.584 (0.455–0.712)
	Malay	154.41	**0.621 (0.515–0.726)**
**hsCRP (μg/ml)**	Caucasian	2.40	**0.589 (0.538–0.641)**
	Chinese	1.69	0.547 (0.481–0.613)
	Indian	8.95	0.550 (0.425–0.675)
	Malay	3.04	0.502 (0.395–0.608)
**CysC (μg/ml)**	Caucasian	0.74	**0.555 (0.501–0.609)**
	Chinese	0.86	0.527 (0.460–0.594)
	Indian	0.85	0.602 (0.475–0.728)
	Malay	0.60	0.571 (0.466–0.677)
**MPO (ng/ml)**	Caucasian	30.17	**0.601 (0.549–0.653)**
	Chinese	36.84	0.532 (0.467–0.598)
	Indian	22.11	0.525 (0.405–0.644)
	Malay	33.40	**0.611 (0.508–0.715)**
**hsTnI (ng/l)**	Caucasian	116.65	**0.618 (0.566–0.671)**
	Chinese	60.75	**0.605 (0.540–0.669)**
	Indian	6.65	0.614 (0.499–0.729)
	Malay	17.15	**0.711 (0.619–0.804)**

Results from ROC analysis, stratified by ethnicity. The optimal cut-off corresponds to the biomarker level at which the largest sum of sensitivity + specificity is found. The AUC is presented with its 95 % confidence interval. When the confidence interval does not contain 0.5 (printed in bold), the cut-off of that biomarker is significantly associated with a SYNTAX score ≥ 18 points.

*ROC* receiver operating characteristics, *AUC* area under the curve, *NTproBNP* N-terminal brain natriuretic peptide, *hsCRP* high-sensitivity C-reactive protein, *CysC* cystatin C, *MPO* myeloperoxidase, *hsTnI* high-sensitivity troponin I.

The optimal cut-off for hsTnI was strikingly higher for Caucasians than the other ethnic groups (116.65 ng/l vs. 60.75 in Chinese, 6.65 in Indians and 17.15 in Malays). This indicates that especially in Indians and Malays, lower levels of hsTnI concur with more severe CAD.

## Discussion

We observed inter-ethnic differences in biomarkers related to CAD. Also, the optimal cut-off levels at which these biomarkers offered discrimination of severe CAD varied substantially between ethnicities.

Differences in NTproBNP levels in the general population have mainly been reported between Blacks and Caucasians, with lower levels reported for Blacks [24]. In our cohort we find the highest fully adjusted levels for Malays and very high cut-off levels were found for Indians and Malays, indicating that in those ethnic groups NTproBNP levels correspond to less severe CAD than in Chinese and Caucasians.

High-sensitivity CRP levels are known to differ markedly among the ethnic groups in the general population with very low levels reported for Chinese and Japanese people [5, 25]. In a Singaporean cohort of individuals visiting the hospital for regular health checks, higher hsCRP levels were found in Indians as compared with Chinese, also when adjusted for confounders [26]. Notably, within the general US population, hsCRP levels were not able to differentiate between US Chinese with and without a future cardiovascular event, while some predictive power was observed for Caucasians, indicating different discriminating properties [25]. Similar to the general population cohorts, high adjusted hsCRP levels were found for Indians and Malays in the current study of CAD patients. In contrast to the general population cohorts, however, we also found higher hsCRP levels in Chinese than in Caucasians in our cohort (albeit not significant after adjustment for confounders). While cut-offs for Caucasians, Chinese and Malays were quite comparable, the hsCRP cut-off for severe CAD in Indians was strikingly higher, suggesting that high hsCRP levels correspond to less severe CAD in Indians than in the other ethnic groups.

In our study we found no inter-ethnic differences in CysC levels among CAD patients whilst inter-ethnic differences have been described in the general population, with lower levels in Blacks than in Whites [27]. To our knowledge, no study has evaluated the ethnicity-specific association of CysC with CAD. The Multi-Ethnic Study of Atherosclerosis (MESA) [28] has evaluated the association of CysC-estimated glomerular filtration rate with the incidence of coronary artery calcium and found a strong association; unfortunately no interactions of this association with ethnicity were tested. In another MESA sub-study [29], however, the association of CysC levels with known biomarkers of CAD (CRP, interleukin-6, intercellular adhesion molecule-I and factor VIII) differed significantly by ethnicity. This leaves the ethnicity-specific role of CysC in relation to CAD severity unresolved.

MPO levels have been reported to be related to coronary atherosclerosis in Blacks, but not in Whites or Hispanics [30], demonstrating an important modifying role of black ethnicity on the association of MPO levels with CAD. No previous comparison of the association of MPO with CAD severity has been made between Asians and Caucasians. In our study we found a significantly stronger association of MPO levels with SYNTAX score among Malays than among Caucasians. However, comparable cut-off levels for all ethnic groups were calculated. The association of MPO with severity of CAD among Malay patients deserves the attention of further research.

A striking difference was found for hsTnI levels, which were much higher in Malays (multivariably adjusted) than in Caucasians. Population levels have been reported to be comparable among Chinese, Indians and Malays in a Malaysian study [31], thus the high levels we observe in Malays can probably not be explained by higher baseline levels. High-sensitivity TnI levels can be elevated without actual myocardial necrosis [32]. Experimental data suggest that hsTnI can be released from cardiomyocytes by release of proteolytic degradation products, which can already occur with mild ischaemia. Mechanical stretch and ischaemia have also been demonstrated to increase cellular wall permeability, leading to leakage of troponins from the cytosol. Lastly, cardiomyocyte-derived vesicles, shed upon ischaemia, might be involved in the release of hsTnI without cardiomyocyte necrosis. These phenomena might explain the high hsTnI levels in stable Malay patients, in the absence of necrosis (Table 2).

Cut-off levels of hsTnI for high SYNTAX score were much higher in Caucasians than in the other ethnic groups, indicating that cardiomyocyte damage is greater in Caucasians than in the other ethnic groups at a SYNTAX score of 18 points. One of the explanations for this feature could be preconditioning occurring in more severe, unstable CAD resulting in resilient myocardium releasing less troponin upon prolonged ischaemia [33]. The Caucasian ethnic group most often presented with stable CAD, thus cardiomyocyte preconditioning would have occurred least in this group.

In summary, our results suggest that inter-ethnic differences in biomarker levels exist and that the association of these biomarkers with the extent of disease differs by ethnicity. Prior to implementation of biomarkers into clinical practice, their performance should be evaluated in an ethnicity-specific manner [34].

### Limitations

Biomarker levels in our study were measured in arterial blood; these levels may differ from venous levels. However, Martin et al. showed that arterial and venous levels of hsCRP and NTproBNP did not differ [35]. Also, levels of NTproBNP, hsCRP, CysC and MPO were measured using an in-house ELISA method instead of clinically standardised assays. Therefore, generalisability of the biomarker levels described in our cohort is limited. Future studies should focus on examining inter-ethnic differences in biomarker levels measured in venous blood, measured by clinically validated assays.

Although follow-up data for all-cause death were available, an ethnicity-specific mortality analysis was not possible due to small numbers of fatalities in certain ethnic groups (e.g. *n* = 3 in the Indian ethnic group). Follow-up data on other adverse cardiovascular events were unfortunately not available.

We have adjusted our results for baseline differences among the ethnic groups as much as possible. However, profound differences were observed at baseline, which might not have been completely abolished after adjustment. In larger multi-ethnic cohorts propensity-matched [36] analysis might be considered in order to further eliminate confounding. Also, details on nutrition, lifestyle and socioeconomic factors were not available in this cohort; therefore we could not consider these (possibly confounding) factors in the analyses.

## Conclusion

In a patient group undergoing coronary angiography we found inter-ethnic differences in levels of biomarkers, related to CAD. These differences persisted after correcting for baseline differences among ethnicities. Furthermore, certain biomarkers displayed inter-ethnic differences in the relationship of biomarkers to severity of CAD, with cut-off values from ROC analyses varying over a five-fold range between ethnicities.

Future research should focus on ethnicity-specific cut-off values of established CAD biomarkers.
